# Supplementary material for: Phosphorylcholine Monoclonal Antibody Therapy Decreases Intraplaque Angiogenesis and Intraplaque Hemorrhage in Murine Vein Grafts
Source: Int J Mol Sci. 2022 Nov 7;23(21):13662. doi: 10.3390/ijms232113662 (PMC9653839; doi:10.3390/ijms232113662)
Supplement: Supplementary file 1 [file ijms-23-13662-s001.zip › ijms-1985620-supplementary.pdf]

International Journal of *Molecular Sciences*

MDPI

Supplementary Materials

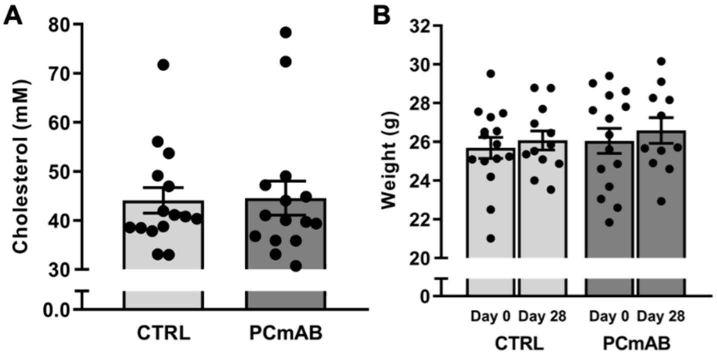

Figure S1. PC-mAb does not affect bodyweight or cholesterol levels. PlasmaSupplementary cholesterol level (A) and bodyweight (B). Data presented as mean ± SEM.

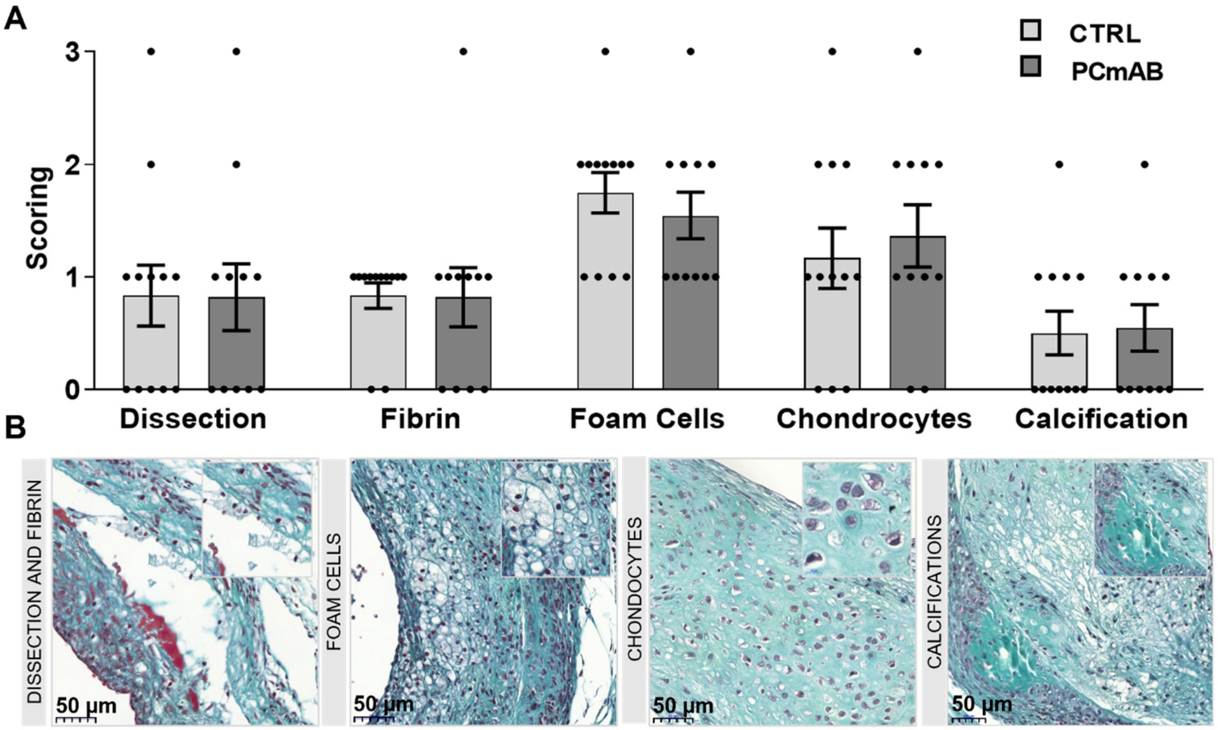

Figure S2. PC-mAb does not alter plaque features. Semi quantitative scoring (A) and representative images of plaque features (B). Data presented as mean ± SEM.

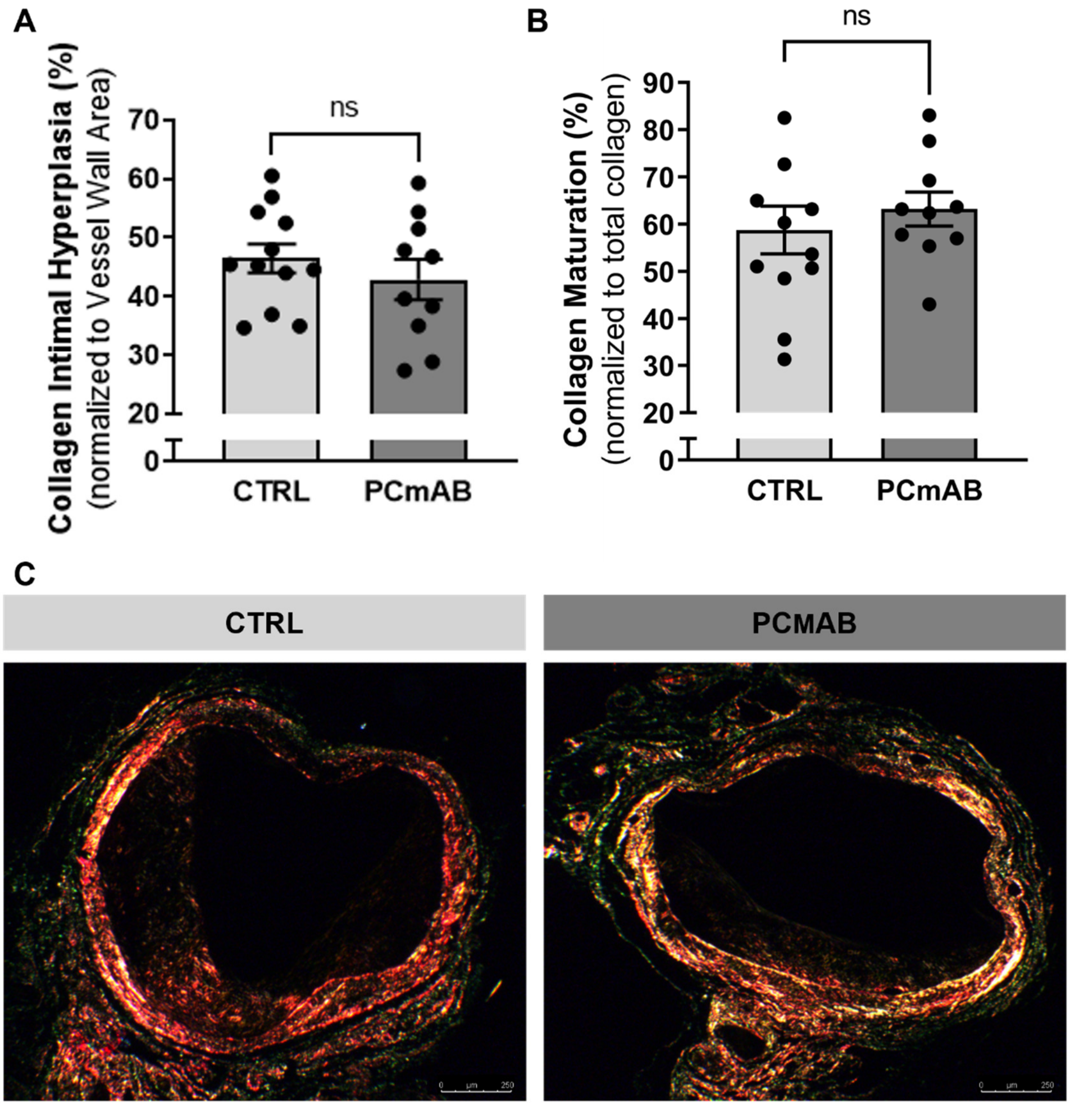

Figure S3. PC-mAb does not alter collagen content in the intima or collagen fiber maturation. Quantification of collagen content in the intimal layer of the vessel wall (A) and quantification of collagen fiber maturation by polarized light (B). Representative images of collagen maturation by polarized light (C). Data presented as mean ± SEM. NS indicates non-significant by t-test.
